# Calcified Fibrous Tumor of Jejunum: A Rare Case Report

**DOI:** 10.7759/cureus.38230

**Published:** 2023-04-27

**Authors:** Divyansh Dwivedi, Ramteja Inturi, Sudhir Jayakar, Prabhat Nichkaode

**Affiliations:** 1 General Surgery, Dr. D. Y. Patil Vidyapeeth, Pune, IND; 2 General Surgery, Dr. D. Y. Patil Vidyapeeth, pune, IND

**Keywords:** leiomyoma, pseudotumor, jejunum, calcified fibrous tumor, mesenchymal tumor

## Abstract

A calcifying fibrous tumor (CFT) is a benign fibroblastic tumor of soft tissues occurring at all ages with no gender predilection. Earlier, it was called a pseudotumor. It may or may not present with symptoms. It can occur anywhere in the body - the most common sites are the stomach, pleura, and intestines. Our study is presented as a case of Intussusception in a young male with symptoms of pain, abdomen, and nausea. The patient underwent an excision of the tumor, and the tumor was examined histo-pathologically and immunohistochemically, showing spindle-shaped cells in dense collagenous tissue with mild inflammation. In this case, a study we are explaining the Clinical and morphological features of the CFT and how to differentiate it from other mesenchymal tumors.

## Introduction

A calcifying fibrous tumor (CFT) is An uncommon benign mass lesion that was initially identified by Rosenthal [1] and Abdul-Karim as a "childhood fibrous tumor with psammoma bodies. Fetsch and colleagues published a more extensive series, redesignating the lesion as "calcifying fibrous pseudotumor [2].

It comprises abundant dense well-circumscribed hyalinized collagen with lymphoplasmacytic infiltrate, spindle cells, lymphoid aggregates, and psammomatous or dystrophic calcifications. Only 12 of the roughly 70 CFTs documented involve the gastrointestinal tract, making it uncommon [3]. It is, therefore, likely to be mistaken for more common spindle cell lesions of the gastrointestinal tract, particularly desmoid tumors and gastrointestinal stromal tumors (GISTs), whose diagnoses might have significant and distinct therapeutic implications. Clinical signs, imaging studies, and endoscopic findings are non-specific. We present a rare case of CFT of jejunum presented as jejuno-jejunal intussusception with signs of intestinal obstruction.

## Case presentation

A 26-year-old patient came to the emergency department complaining of pain in the abdomen for one month, complaints of constipation, and complaints of vomiting within 15- 30 minutes of food consumption for one month. Vomiting was non-projectile containing food particles as content, and which got aggravated for two days.

The patient was evaluated clinically -and on examination, revealed diffuse abdominal tenderness; his dehydration was corrected, and an x-ray of the erect abdomen showed dilated bowel loops. Ultrasonography was done, which suggested an 11.0X3.7 cm oblong mass in the left iliac and hypo-gastric region consistent with small bowel intussusception. Routine clinical investigations were done, as shown in Table 1. 

**Table 1:** Clinical investigations TLC: Total Leukocyte Count.

Investigations	
Haemoglobin	10.9g/dl
TLC	6000/ul
Platelet count	398000/ul
Bilirubin total	0.32mg/dl
Total Protein	6.7gm/dl
Serum Albumin	3.8gm/dl
Serum Globulin	2.9gm/dl
Urea	20mg/dl
Serum Creatinine	0.63mg/dl
Sodium	mmol/lit
Potassium	mmol/lit
Immuno Histochemically	
S 100	Negative
Small Muscle Actin	Negative
CD117 (KIT)	Negative

**Figure 1:**
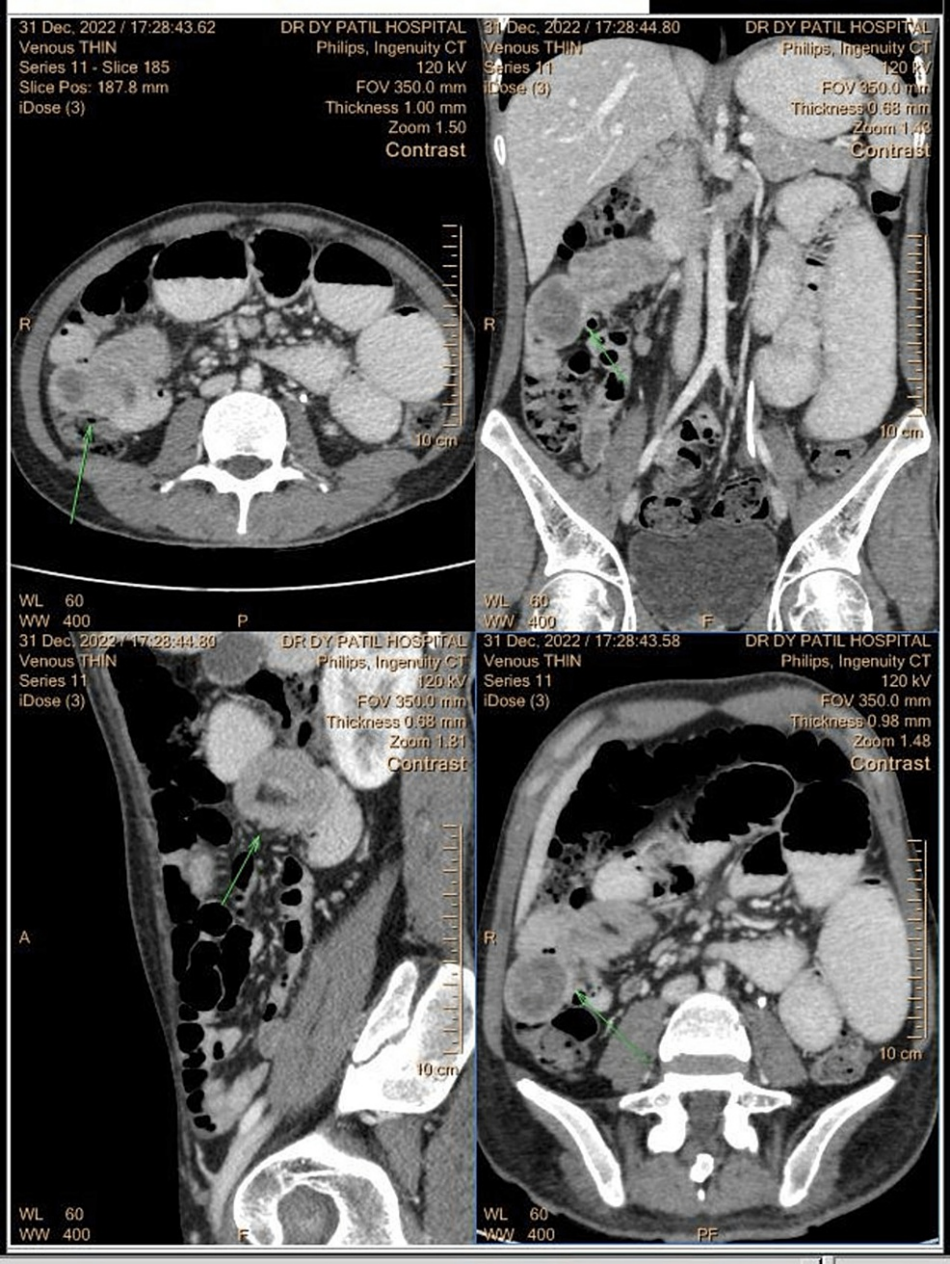
CECT Abdomen showing moderate to severe dilatation of jejunum due to hypodense enhancing amorphous lesion arising from jejunum with jejuno-jejunal intussusception (marked with green arrow). CECT: contrast-enhanced computed tomography

Contrast-enhanced computed tomography (CECT) of the abdomen suggested moderate to severe dilatation of the jejunum due to hypodense enhancing amorphous mass lesion arising from the jejunum with jejuno-jejunal intussusception, and the mass showed mild to moderate enhancement and likely polypoidal mass leading to intussusception (Figure 1). The patient was adequately resuscitated and was taken for exploratory laparotomy under general anesthesia. The intraoperative findings showed evidence of jejuno-jejunal intussusception notes approximately 15 cm distal to the duodenojejunal flexure.

**Figure 2:**
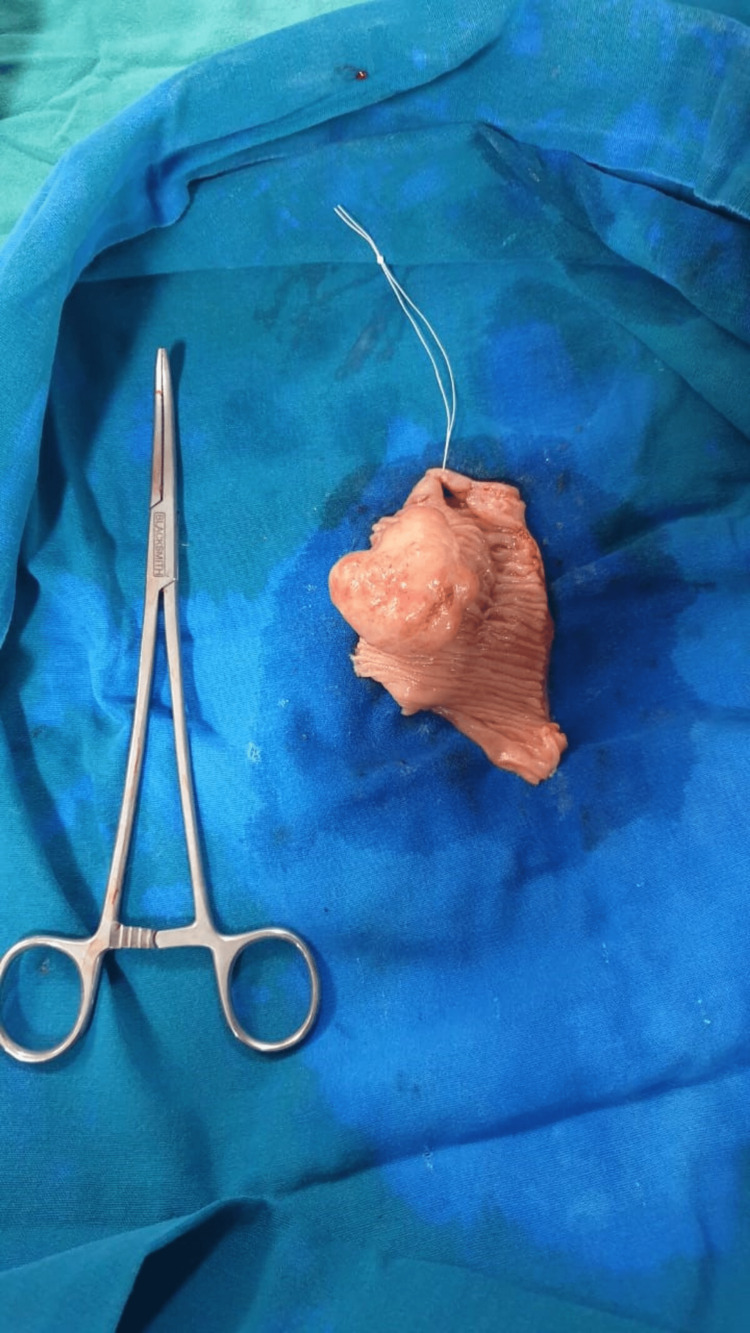
Excised specimen

Distal to the intussusception, the small bowel appears collapsed, intussusception was manually reduced, a 4x5cm firm mass noted intra luminally arising from the jejunal wall, resection, and anastomosis with a 2 cm margin was done, and the specimen was sent for histopathological examination (Figure 2). Histopathologically a pedunculated grey-white polypoidal mass was noted on the mucosal surface measuring 5x4x4cm.

**Figure 3:**
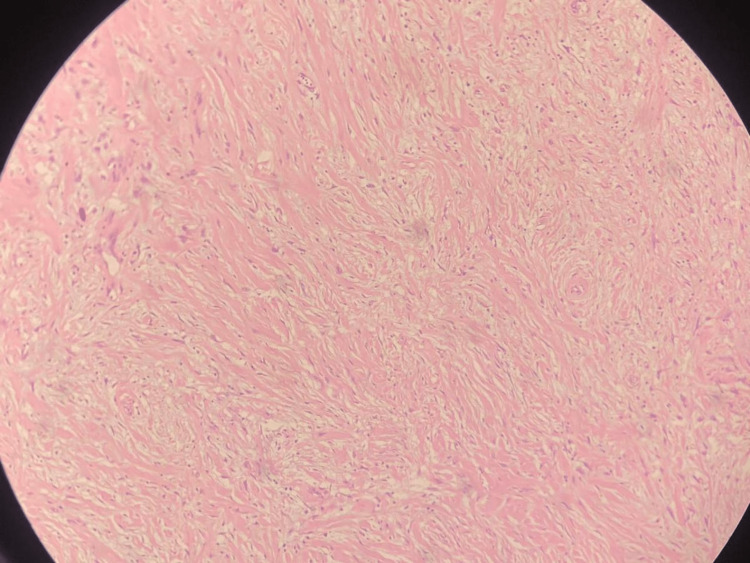
Photomicrograph showing bland spindle-shaped cells in dense collagenous tissue with mild mixed inflammation (H&E, 400X).

Micro-multiple sections studied show pauci-cellular fibroblastic proliferation of spindle-shaped cells embedded in dense collagenous tissue, there is a varying degree of lymphocytes and plasma cells, eosinophils with small foci of dystrophic/psammatous calcification (Figure 3).

## Discussion

Adult intussusception makes up just 1-5% of adult intestinal obstructions and accounts for 5% of all intussusception occurrences [4]. The majority of adult intussusceptions are secondary intussusceptions having a lead point. The diagnosis of the said condition is usually helped by CECT [5]. The intra-luminal masses identified in such conditions are benign (Stromal Tumors, Lipomas, Hamartomas, Schwannoma, Leiomyoma) and malignant (neuroendocrine tumors, adenocarcinomas, lymphoma, malignant GIST). Small bowel cancer is uncommon, i.e., less than 5% of gastrointestinal cancers, despite it (small intestine) making up 75% of the digestive tract and 90% of its mucosal surface area [6].

Intestinal obstruction associated with intussusception is an emergency that needs immediate exploration due to its dire sequelae of complications (3rd space loss leading to dehydration, bacterial translocation leading to septicemia, increased intra luminal pressure leading to perforation peritonitis). Plain abdominal radiographs are usually diagnostic of bowel obstruction in up to 86% of the cases, but in 20-30% of cases, further imaging (like CT or barium radiography) may be required. CECT aids in the identification of the location of the transition point, likely pathology.

Based on the Clinical, Radiological, and Intra-operative Findings, we suspected the mass to be jejune-jejunal GIST leading to intussusception; hence R0 resection was done with a 1- to 2-cm macroscopic margin, which is sufficient to achieve microscopically negative margins [7]. Intraluminal CFT in the GI tract is rare, and correct identification is important to distinguish it from other mesenchymal GI lesions. GI tract CFTs are usually asymptomatic but rarely cause serious complications such as intussusception leading to an obstruction like in our case [8]. CFTs are histologically characterized by hyalinized collagen, bland spindle cells, lymphoplasmacytic infiltrates, psammoma bodies, or dystrophic calcifications. GI tract CFTs differ from other spindle cell mesenchymal neoplasms like GIST, Schwannoma, and Leiomyoma.

In contrast to gastrointestinal CFTs, GIST cells are typically more cellular and have fibrillar stroma, whereas CFTs typically have hyaline in the stroma and lymphoplasmacytic infiltration. Spindle cells of GIST are positive for CD117, CD34, and DOG-1 and frequently display somatic KIT mutation [9].

Shwannoma's paucicellularity, regions of hyalinization and calcification, and peripheral lymphoid aggregation can also make them look like CFT. However, schwannomas' spindle cells feature distinctive undulating nuclei and are S100-positive [10]. Leiomyomas can be quite paucicellular, hyalinized, and heavily calcified, yet they vary from CFT due to their fascicular architecture, fusiform tumor cell nuclei, and smooth muscle actin positive [11].

IMT (Inflammatory Myofibroblastic Tumor) is histologically similar to CFT, but the fact that IMT uniformly stains smooth muscle actin differentiates it from CFT [12]. Treatment of CFT is targeted at relieving the symptoms of intussusception /obstruction. Intestinal masses have been predominantly treated by segmental resection with healthy margins.

## Conclusions

CFT has been predominantly considered a deep soft tissue tumor without malignant potential; its occurrence involving the GI tract is rare. Clinical and imaging findings are usually non-specific, and definitive diagnosis can only be made by histopathology and IHC.
